# E-health: Determinants, opportunities, challenges and the way forward for countries in the WHO African Region

**DOI:** 10.1186/1471-2458-5-137

**Published:** 2005-12-20

**Authors:** Joses M Kirigia, Anthony Seddoh, Doris Gatwiri, Lenity HK Muthuri, Janet Seddoh

**Affiliations:** 1 World Health Organization, Regional Office for Africa, Brazzaville, Congo; 2 School of Public Health, University of New South Wales, Australia; 3 School of Public Health, Department of Health Sciences, Kenyatta University, Kenya; 4 West African Examinations Council, Accra, Ghana

## Abstract

**Background:**

The implementation of the 58^th ^World Health Assembly resolution on e-health will pose a major challenge for the Member States of the World Health Organization (WHO) African Region due to lack of information and communications technology (ICT) and mass Internet connectivity, compounded by a paucity of ICT-related knowledge and skills. The key objectives of this article are to: (i) explore the key determinants of personal computers (PCs), telephone mainline and cellular and Internet penetration/connectivity in the African Region; and (ii) to propose actions needed to create an enabling environment for e-health services growth and utilization in the Region.

**Methods:**

The effects of school enrolment, per capita income and governance variables on the number of PCs, telephone mainlines, cellular phone subscribers and Internet users were estimated using a double-log regression model and cross-sectional data on various Member States in the African Region. The analysis was based on 45 of the 46 countries that comprise the Region. The data were obtained from the United Nations Development Programme (UNDP), the World Bank and the International Telecommunications Union (ITU) sources.

**Results:**

There were a number of main findings: (i) the adult literacy and total number of Internet users had a statistically significant (at 5% level in a t-distribution test) positive effect on the number of PCs in a country; (ii) the combined school enrolment rate and per capita income had a statistically significant direct effect on the number of telephone mainlines and cellular telephone subscribers; (iii) the regulatory quality had statistically significant negative effect on the number of telephone mainlines; (iv) similarly, the combined school enrolment ratio and the number of telephone mainlines had a statistically significant positive relationship with Internet usage; and (v) there were major inequalities in ICT connectivity between upper-middle, lower-middle and low income countries in the Region. By focusing on the adoption of specific technologies we attempted to interpret correlates in terms of relationships instead of absolute "causals".

**Conclusion:**

In order to improve access to health care, especially for the majority of Africans living in remote rural areas, there is need to boost the availability and utilization of e-health services. Thus, universal access to e-health ought to be a vision for all countries in the African Region. Each country ought to develop a road map in a strategic e-health plan that will, over time, enable its citizens to realize that vision.

## Background

Information technology (IT) is now viewed globally as a catalyst for change "not only to improve the automated processes but also to improve the way work is performed" [1]. In 1997, the World Health Assembly identified fostering the use of, and innovation in, science and technology for health as one of the essential functions of sustainable health systems [2]. The general orientation then was that the ongoing information and technology revolution (including cellular phones and the Internet boom) will introduce greater fluidity, allowing virtual teams to come together and disband as needed.

The required systems and architecture may lead to the restructuring of health systems organization and support greater external linkages, including strategic alliances or other partnering activities [1]. In 2000, the United Nations General Assembly, in its Millennium Development Declaration, called upon all Member countries to cooperate with the private sector to "make available the benefits of new technologies, especially information and communication" [3]. In May 2005, the 58^th ^World Health Assembly adopted a resolution on e-health calling on all the 192 WHO Member States to leverage the use of e-health in the pursuit of health-for-all vision [4].

Though the stage has been set for engaging this new technology for health, the perimeters for what it means within the health industry are not precise. Oh et al. [5] demonstrate that any query string "ehealth" or "e-health" is likely to turn up bibliographic databases with materials that contain as many definitions as there are articles. Generally, however, e-health encompasses both the use of telecommunications for the diagnosis and treatment of disease and ill-health (i.e. tele-medicine) and the use of computer-assisted ICT to support the management, surveillance, health promotion, health education, health services, health research, access to health-related knowledge and other public health functions (i.e. tele-health) [6].

What is consistent as we review literature is that though there are various 'neologisms', there is a consensus on the benefits of e-health to service delivery. Eysenbach [7] summarizes rather well the potential promises: efficiency, enhanced quality of care, evidence-based e-health interventions, empowerment of e-health service consumers and encouragement of true partnership between the patient and the health professional. The other benefit in our view is that ICT treats geographically dispersed resources as if they were centralized, thus promoting economies of scale, offering time flexibility and responsiveness on the part of pharmacists, doctors, nurses, administrators and health care managers.

There is thus adequate reason for the health industry to reform as the shape of the future of core health services is increasingly determined by ICT. Developed countries are already investing heavily in ICT connectivity and the possibilities it offers. In the United Kingdom, the government is known to have invested £6 200 million in establishing a National Program for Information Technology (NPfIT) in the National Health Service (NHS) [8]. This program aims to deliver electronic records, electronic prescribing and electronic booking of appointments [9]. In individual hospitals across the United States, facilities are developing plans which at the time of initiation may take 10 years to complete just to get clinical and financial data through both wire and wireless technology with a view to ensuring increased management efficiency and improved patient care at dispersed locations [10,11].

However, as Ginter et al. [1, p. 298] noted: "The seamless connectivity to all components of a health delivery system ... (and to) ... providers, employers, payors, pharmacies and regulatory agencies is a technological challenge, but the potential for cost reduction and improvement in the quality of care is significant." Obviously, all nations and health organizations that contemplate exploiting the global e-health opportunities will need to surmount this challenge.

For the African Region, the challenge raises some salient questions. Are the Member countries appropriately positioned, in terms of ICT connectivity, to enjoy these benefits of e-health? What is the state of PCs, telephone and Internet connectivity? What are the main determinants of that connectivity?

The purpose of this paper is to review the current situation and put forward some evidence that could assist policy-makers to think seriously about the critical variables necessary to promote e-health. In this regard we intend to: (i) review the general availability of PCs, telephones and Internet connectivity in the Region; (ii) model the effects of adult literacy, per capita income, regulatory quality, total number of Internet users and total population on the total number of PCs in a country; (iii) model the effects of combined enrolment ratio, per capita income, regulatory quality, rule of law and corruption control on mainline telephone connectivity; (iv) model the effects of combined enrolment ratio, per capita income, regulatory quality, rule of law and corruption control on cellular phone connectivity; (v) model the effects of adult literacy, combined enrolment ratio, per capita income, electricity consumption, PCs per 100 people, fight against corruption and the number of telephone mainlines on Internet connectivity (NET); and (vi) to propose actions needed to create an enabling environment for e-health services growth and utilization in the Region. We also hope that this paper will elicit more debate and research into various aspects of e-health as it relates to the WHO African Region.

## Methods

This is an exploratory analysis of secondary data on the countries in the WHO African Region obtained from the UNDP [12]; the World Bank [13]; and the ITU [14] sources. The WHO African Region consists of 46 Member countries, of which 21 are francophone, 20 are anglophone and five are Portuguese-speaking. The data were then used to estimate three equations contained in the conceptual framework developed below. The whole dataset for one country was not available.

### Conceptual framework

According to Quibria et al. [15], depending on the type of use, ICT can be divided into three categories: (i) computing; (ii) communication; and (iii) Internet-enabled communication and computing. There exist one-way (e.g. radio and television) and two-way (e.g. fax, telephone, telegraph, pager) communications. The Internet's growth is a function of two-way communication link between telephone lines and PCs.

Fixed landline telephones are the traditional means of vital communication within national health systems, and the Internet connectivity in African countries greatly depends on the existence of telephone mainlines. However, since they are often owned by state corporations, their management is often inefficient, which results in making the installation costs and telephone services expensive, and thus beyond the reach of a majority of the people in Africa. In some of the countries that have privatized telecommunication services as part of public sector reform, the cost of telephone services has been decreasing due to competition [12]. In most countries in Africa, mobile telephones are easier to obtain and less prone to corrupt public sector practices than fixed landline telephones. We concur with Quibria *et al*. [15] that the migration of the Internet and Internet applications into mobile phone systems will have tremendous technological implications for e-health practice in developing countries.

In order to explore the statistically significant determinants of PC ownership, telephone mainlines connectivity, cellular phone connectivity and Internet connectivity, four log-log (or double log) models were estimated with the full dataset of the countries in the Region.

Firstly, to model the effects of adult literacy ratio (AL), GDP per capita (Y), regulatory quality (RQ), total number of Internet users (NET) and total population (TPOP) on the total number of PCs, the following regression equation was estimated:

log *PCs *= log α + β_1 _log *AL *+ β_2 _log *Y *+ β_3 _log *RQ *+ β_4 _log *NET *+ β_5 _log *TROP *+ ∈ ....... (1)

Secondly, to model the effects of combined gross enrolment ratio for schools (EN), GDP per capita (Y), regulatory quality (RQ), rule of law (RL) and corruption control (CC) on telephone mainlines per 1 000 people (TML), the following equation was estimated:

log *TML *= log α + β_1 _log *EN *+ β_2 _log *Y *+ β_3 _log *RQ *+β_4 _log *RL *+β_5 _log *CC *+ ∈ ....... (2)

Thirdly, to model the effects of combined gross enrolment ratio for schools (EN), GDP per capita (Y), regulatory quality (RQ), rule of law (RL), and corruption control (CC) on cellular telephone subscribers per 1 000 people (CS), we estimated the following equation:

log *CS *= log α + β_1 _log *EN *+ β_2 _log *Y *+ β_3 _log *RQ *+ β_4 _log *RL *+β_5 _log *CC *+ ∈ ....... (3)

Lastly, to model the effects of combined gross enrolment ratio for schools (EN), per capita income (Y), electricity consumption (EC), corruption control (CC) and the number of telephone mainlines (TML) on Internet connectivity (NET), we estimated the equation:

log *NET *= log α + β_1 _log *EN *+ β_2 _log *Y *+ β_3 _log *EC *+ β_4 _log *CC *+ β_5 _log *TML *+ ∈ ................................................. (4)

where: log is the natural logarithm (i.e. log to the base e, where e equals 2.718);α is the intercept term; β's are the coefficients of elasticity, which can take any value between 0 (perfectly inelastic) to ∞ (perfectly/infinitely elastic); and ∈ is a stochastic (random) error term capturing all factors that affect, for example, the number of telephone mainlines, cellular telephone subscribers and Internet users (in a country) but are not taken into account explicitly in each of the four models.

Thus, for example, in equation 1: (i) α (the intercept term) refers to the number of total PCs if all the explanatory variables included in the model were equal to zero; (ii) the slope coefficient measures the elasticity (responsiveness) of dependent variable PCs with respect to explanatory variables (AL, Y, RQ, NET, TPOP), that is, the percentage change in PCs for a given small percentage change in one explanatory variable, while holding the others constant.

Since theory does not provide much guidance on model specification, the choice of explanatory variables in the current study were guided by the past telecommunication demand studies. The coefficients of the variables included in equations were a priori expected to assume the signs indicated in Table 1, 2, 3, 4.

**Table 1:** Hypothesized relationships between the personal computer penetration and independent variables in equation 1

**Independent variables**	**Variable coefficient**	**Expected Sign**	**Studies from which the hypothesized signs are based**
Adult literacy (Education)	β_1_	Positive	Valleta & MacDonald [16];Quibria et al [15];Chinn MD & Fairlie [17];Dewan et al [18]
Per capita income	β_2_	Positive	Valleta & MacDonald [16];Quibria et al [15];Kiiski & Pahjola [19];Dewan et al [18]
Regulatory quality	β_3_	Positive	Chinn MD & Fairlie [17]
Total number of internet users	β_4_	Positive	Kiiski & Pahjola [19];
Total population	β_5_	Positive	Chinn MD & Fairlie [17]

**Table 2:** Hypothesized relationships between the number of telephone mainlines per 1000 people and independent variables in equation 2

**Independent variables**	**Variable coefficient**	**Expected Sign**	**Studies from which the hypothesized signs are based**
Combined school enrolment ratio	β_1_	Positive	Quibria et al [15];Chinn MD & Fairlie [17]
Per capita income	β_2_	Positive	Oyelaran-Oyeyinka & Lal [21];
Regulatory quality	β_3_	Indeterminate	
Rule of law	β_4_	Positive	
Corruption control	β_5_	Indeterminate	

**Table 3:** Hypothesized relationships between the number of cellular phone subscribers per 1000 people and independent variables in equation 3

**Independent variables**	**Variable Coefficients**	**Expected Signs**	**Studies from which the hypothesized signs are based**
Combined school enrolment ratio	β_1_	Positive	Chinn & Fairlie [17];
Per capita income	β_2_	Positive	Chinn & Fairlie [17];Oyelaran-Oyeyinka & Lal [21];Quibria et al [15]
Regulatory quality	β_3_	Positive	Chinn & Fairlie [17];
Rule of law	β_4_	Indeterminate	
Corruption control	β_5_	Indeterminate	

**Table 4:** Hypothesized relationships between the number of internet user per 1000 people and independent variables

**Independent variables**	**Variable Coefficient**	**Expected Sign**	**Studies from which the hypothesized signs are based**
Combined school enrolment ratio	β_1_	Positive	Chinn MD & Fairlie [17]; Muller [22]; Dewan et al [18]; Comin & Hobijn [20]
Per capita income	β_2_	Positive	Kiiski & Pahjola [19]; Quibria et al [15]; Chinn & Fairlie [17]; Muller [22]; Dewan et al [18]; Comin & Hobijn [20]
Electricity consumption per person	β_3_	Positive	Chinn MD & Fairlie [17]
Telephone mainlines per 1000 persons	β_4_	Positive	Oyelaran-Oyeyinka & Lal [21]; Chinn & Fairlie [17]; Muller [22]; Dewan et al [18]
Corruption control/Regulatory quality	β_5_	Positive	Chinn & Fairlie [17]; Muller [22]
Cellular phone users	β_6_	Positive	Muller [22]

The raw data were entered into the computer using the EXCEL spreadsheet program and subsequently exported to STATA [23] software for statistical analysis. In order to estimate the double-logarithmic equations, standard STATA commands were used to transform the dependent and independent variables into their logarithms. All the dependent and independent variables are defined in Table 5.

**Table 5:** Definition of variables and sources of data

**Variable**	**Variable description**	**Sources of data**
AL	Adult literacy rate (% ages 15 and above)	UNDP [12]
ER	Combined gross enrolment ratio for primary, secondary and tertiary schools (%)	UNDP [12]
Y	GDP per capita, expressed in international dollars (in $PPP)	UNDP [12]
EL	Electricity consumption per capita (kilowatt-hours)	UNDP [12]
PCs	Personal computers (PCs) per 100 people	ITU [14]
TPCs	Total number of PCs in a country	UNDP [12]
TML	Telephone mainlines per 1 000 people	ITU [14]
CS	Cellular subscribers per 1 000 people	ITU [14]
NET	Number of internet users per 1 000 people	ITU [14]
RQ	Regulatory quality: measured in units ranging from -2.5 to 2.5, with higher values corresponding to better governance outcomes	World Bank [13]
RL	Rule of law: measured in units ranging from -2.5 to 2.5, with higher values corresponding to better governance outcomes	World Bank [13]
CC	Corruption control: measured in units ranging from -2.5 to 2.5, with higher values corresponding to better governance outcomes	World Bank [13]
TPOP	Total human population in a country	UNDP [12]

## Results

### Descriptive statistics

Table 6 presents the mean, standard deviation and minimum and maximum values for various dependent and independent variables. On average, countries in the African Region have 32 telephone mainlines, 60 cellular subscribers and 17 Internet users per 1 000 people and two PCs per 100 people. Thirty-seven (82%) countries have 41 telephone mainlines per 1 000 people; 29 (64%) countries have less than 41 cellular subscribers per 1 000 people; and 39 (87%) countries have less than 41 Internet users per 1 000 people. The Figure shows the frequency distribution of countries by various brackets of the number of telephone mainlines, cellular subscribers and Internet users per 1 000 people. The average adult literacy rate is 61%, net primary enrolment ratio is 68%, net secondary education enrolment ratio is 29% and combined school enrolment rate is 50%.

**Table 6:** Descriptive statistics for dependent and independent variables

**Variables**	**No. of countries**	**Mean**	**Standard deviation**	**Minimum**	**Maximum**
Adult literacy rate	45	60.95	20.79	12.8	91.9
Combined primary, secondary and tertiary enrolment rate	45	49.67	17.04	19	85
Net primary enrolment ratio	39	68.03	20.52	30	106
Net secondary enrolment ratio	29	28.69	21.93	5	98
Percapita income ($PPP)	44	3158.39	5276.94	520	30 13
Electricity consumption per capita	37	151.30	214.49	12	950
Total number of PCs	45	192102.6	537485.80	4000	3300000
Telephone main lines per 1000 people	45	31.58	60.42	0	270
Cellular subscribers per 1000 people	45	60.29	104.79	0	553
Internet users per 1000 people	45	16.87	28.83	0.7	145.2
Regulatory quality	46	-0.62	0.64	-2.15	0.96
Rule of law	46	-0.72	0.61	-1.76	0.84
Corruption control	46	-0.62	0.52	-1.65	0.86
Population	45	15100000	22300000	80000	123000000

### Determinants of PCs in a country

Table 7 shows the results of regression of logarithm of total number of PCs against logarithms of adult literacy (AL), per capita income (Y), regulatory quality (RQ), total number of Internet users (NET) and total population (TPOP) (i.e. *equation 1*). These five variables explain 91% of the variations in the number of PCs in a country. The regression coefficients of the AL and NET had a positive sign and were statistically significant at 5% level of significance. Thus, it is clear that as adult literacy and the number of Internet users grows the demand for PCs also increases.

**Table 7:** Regression of logarithm of the number of personal computers (PCs)

**Variable**	**Coefficient**	**t-statistic**	**P > t**	**95% Conf. Interval**
Logarithm of adult literacy	0.435	2.24*	0.032	0.039 to 0.831
Logarithm of per capita income	0.194	1.73	0.093	-0.0338 to 0.421
Regulatory quality	0.142	1.13	0.269	-0.115 to 0.399
Logarithm of total number of Internet users	0.804	9.56*	0.000	0.633 to 0.975
Logarithm of total population	0.143	1.47	0.150	-0.054 to 0.339
Constant	-3.291	-1.88	0.069	-6.853 to 0.272
Number of observations	39			
F(5, 33)	75.69			
Prob > F	0.000			
Adjusted R-squared	0.908			

*Statistically significant at 95% level of confidence. This means that the computed t-value (t_k_) is greater than the critical t-value (t_c_) of 2.042 which was obtained from a table of critical values of the t-distribution.

### Determinants of number of telephone mainlines in a country

Table 8 presents the results of regression of telephone mainlines per 1 000 people against combined school enrolment ratio (EN), per capita income (Y), regulatory quality (RQ), rule of law (RL) and corruption control (CC) (i.e. *equation 2*). These five variables explain about 73% of the variations in the number of telephone mainlines in a country. The regression coefficients of the EN and Y had a positive sign and were statistically significant at 5% level of significance. Thus, it is clear that as combined school enrolment and per capita income grows, the telephone mainline connectivity improves. The regulatory quality (governance proxy) took a negative sign and was statistically significant at 5% level of significance.

**Table 8:** Regression of logarithm of the number of telephone mainlines per 1 000 people

**Variable**	**Coefficient**	**t-statistic**	**P > t**	**95% Conf. Interval**
Logarithm of combined enrolment ratio	0.911	2.63*	0.012	0.306 to 0.839
Logarithm of per capita income	0.573	4.36*	0.000	0.306 to 0.839
Regulatory quality	-0.791	-2.71*	0.010	-1.384 to -0.199
Rule of law	0.777	1.67	0.102	-0.163 to 1.717
Corruption	0.709	1.73	0.091	-0.119 to 1.538
Constant	-4.852	-3.79	0.001	-7.444 to -2.260
Number of observations	43			
F(5, 37) =	23.20			
Prob > F	0.000			
Adjusted R-squared	0.726			

*Statistically significant at 95% level of confidence.

From the results in Table 9 it is seen that the combined education enrolment elasticity coefficient is 0.91, implying that for a 1% increase in combined education enrolment, the availability of telephone mainlines on average increases by about 0.91%. Since the combined education enrolment elasticity coefficient is less than one in absolute terms, it can be said that the availability of telephone mainlines was education-inelastic (i.e. not very responsive to education). A similar pattern is observed for the remaining explanatory variable coefficients.

**Table 9:** Regression of logarithm of the number of cellular subscribers per 1 000 people

**Variable**	**Coefficient**	**t-statistic**	**P > t**	**95% Conf. Interval**
Logarithm of combined enrolment ratio	1.448	3.05*	0.004	0.485 to 2.411
Logarithm of per capita income	0.601	3.33*	0.002	0.234 to 0.969
Regulatory quality	0.144	0.35	0.732	-0.705 to 0.993
Rule of law	-0.616	-0.92	0.363	-1.972 to 0.740
Corruption	0.992	1.71	0.095	-.183 to 2.167
Constant	-6.656	-3.74	0.001	-10.271 to -3.041
Number of observations	41			
F(5, 35) =	13.43			
Prob > F	0.0000			
Adjusted R-squared	0.6084			

*Statistically significant at 95% level of confidence.

### Determinants of number of cellular phone subscribers in a country

The results obtained from the estimation of *equation 3 *are summarized in Table 10. The five explanatory variables (EN, Y, RQ, RL and CC) explain almost 61% of the variations in the number of cellular phone subscribers in a country. The combined school enrolment ratio (EN) and per capita income (Y) have a statistically significant (at 5% level) positive effect on the number of cellular subscribers in a country. Thus, as the per capita income and school enrolment increases, the number of people with capability of subscribing to cellular phone grows.

**Table 10:** Regression of logarithm of the number of internet user per 1 000 people

**Variable**	**Coefficient**	**t-statistic**	**P > t**	**95% Conf. Interval**
Logarithm of combined enrolment ratio	0.906	2.13*	0.042	0.036 to 1.771
Logarithm of per capita income	0.007	0.04	0.969	-0.389 to 0.374
Log of electricity consumption	0.051	0.43	0.673	-0.195 to 0.297
Logarithm of telephone mainlines per 1000 persons	0.622	3.60*	0.001	0.268 to 0.976
Corruption control	0.297	0.89	0.381	-0.386 to 0.981
Constant	-3.130	-1.84	0.077	-6.619 to 0.359
Number of observations	34			
F(5, 35) =	16.77			
Prob > F	0.0000			
Adjusted R-squared	0.705			

*Statistically significant at 95% level of confidence.

From the results in Table 10 it is seen that the combined school enrolment elasticity coefficient is 1.45, implying that for a 1% increase in combined school enrolment, the cellular phone subscription on average increases by about 1.5%. Since the combined education enrolment elasticity coefficient is greater than one in absolute terms, it can be said that the cellular phone subscription is education-elastic (i.e. very responsive to education). On the other hand, the per capita income elasticity value was 0.6. This is less than one in absolute terms, which indicates that the cellular subscription is income-inelastic, i.e. a unit increase in per capita income elicits a less than proportionate increase in cellular phone subscription.

### Determinants of number of Internet users in a country

Table 11 portrays the results from the estimation of *equation 4*. Since the computed F-statistic is greater than the critical F-statistic at 5% level of significance, we can conclude that the model has a good fit. This is also supported by the adjusted R-squared of 0.70, which means that the five explanatory variables included in *equation 4 *explain about 70% of the total variations in the number of Internet users in a country.

**Table 11:** Total number of internet hosts, internet users, PCs, cellular subscribers, telephone mainlines, telephone subscribers across various income groupings of countries in the African region

**Variable**	**Upper-middle income countries (N = 4)**		**Lower-middle income countries (N = 5)**		**Low income countries (N = 36)**	
			
	**Total number**	**Number per 1 000 people**	**Total number**	**Number per 1 000 people**	**Total number**	**Number per 1 000 people**
Total internet hosts	6452	1.47	294127	3.61	628470.6	1.06
Total internet users	256700	58.34	3712000	45.52	3739500	6.32
Total personal computers	293000	66.59	3821000	46.85	3378000	5.71
Total cellular subscribers	1198000	272.27	18666400	228.90	15255000	25.80
Total telephone mainlines	539600	122.64	7288900	89.38	4017300	6.79
Total telephone subscribers	1737700	394.93	22797300	279.55	17937400	30.33
Population	4400000		81550000		591340000	

Notes: Low income – economies with gross national income (GNI) per capita of US$825 or less; Lower-middle income – economies with a GNI per capita of more than US$826 and less than US$3255; Upper-middle income – economies with a GNI per capita of more than US$3256 and less US$10065.

The regression coefficient of the combined school enrolment ratio (EN) and the number of telephone mainlines per 1000 persons (TML) were positive and statistically significant at 5% level of significance. Therefore, as combined education enrolment and telephone mainlines increased, Internet use improved.

Since the elasticity coefficient for combined school enrolment was less than one (0.906), it implied that a one percentage point increase in school enrolment could lead to a less than proportionate increase in the Internet usage.

## Discussion

The low combined school enrolment, high illiteracy rates, low per capita incomes, widespread poverty and weak ICT connectivity pose a major challenge to most of the African countries' efforts to leverage the global opportunities provided by e-health. Among several other possible strategies for promoting the use of ICT in the pursuit of public health objectives, countries have the choice to pursue holistic economic growth strategies that increase per capita incomes or to invest specifically in increasing education enrolment.

From our analysis so far, we are of the opinion that the relatively low average adult literacy rate of 61%, net primary enrolment ratio of 68%, net secondary education enrolment ratio of 29% and combined school enrolment rate of 50% are likely to hamper effective telecommunication growth and utilization of e-health services. Per capita income and formal education appears to have significant effect on telecommunication usage. This expresses the need for increasing school enrolment as a key factor in telecommunication usage.

Generally, a strategy geared at increasing the primary, secondary and tertiary education enrolment is likely to have a positive impact on the usage of telephone mainlines.

**Figure 1:**
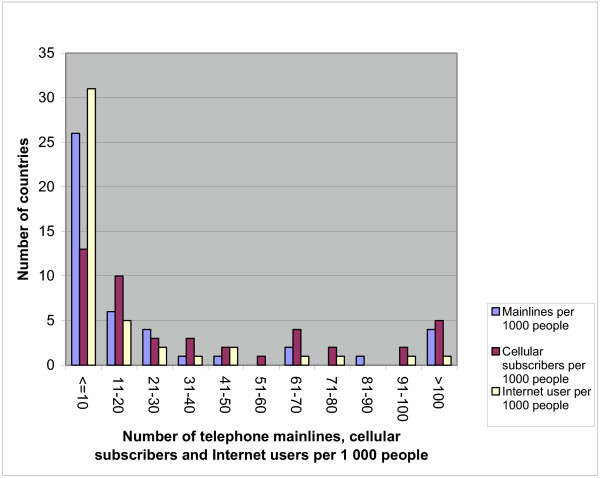
Distribution of countries by ICT.

When countries in the African Region are making a choice of strategies to tap into the e-health-related opportunities, it will be important to take cognizance of the fact that there are major ICT connectivity inequalities across the various income groupings of the countries. Indeed, the ICT connectivity and use varies from country to country (see Figure 1) and from one income grouping of countries to another (Table 11). For example, there are: (i) 67 PCs per 1 000 people among upper-middle income countries compared to 6 PCs per 1 000 people in low income countries; (ii) 123 telephone mainlines per 1 000 people among upper-middle income countries compared to 7 telephone mainlines per 1 000 people in low income countries; (iii) 272 cellular phone subscribers per 1 000 people among upper-middle income countries compared to 26 cellular phone subscribers per 1 000 people in low income countries; (iv) 395 total telephone subscribers per 1 000 people among upper middle income countries compared to 30 total telephone subscribers per 1000 people in low income countries; and (v) 58 Internet users per 1 000 people among upper-middle income countries compared to 6 Internet subscribers per 1 000 people in low income countries.

The advent of the cellular telephone presents an important opportunity for the practice of e-health in the African Region. It has a number of important attributes: (i) voice communication; (ii) written text communication through SMS; (iii) new generation of cellular phones have e-mail facility; and (iv) even in the absence of telephone mainlines, cellular phones and satellites can enable Internet connectivity.

The question of regulatory quality is important for technological growth. For instance, Moshin and Ishaq [24] noted that countries with state controlled telecommunications monopolies and the absence of competitive forces impended the growth, operational expansion, and induction of new technologies into the telecommunications arena. Comin and Hobjin [20,p.3] also observed that "in terms of political institutions, countries where the executive power is in the hands of the military or of an agent that does not hold any public position, tend to adopt technologies more sluggishly". From this perspective, in economies where the private sector plays a significant role, it is known that competition has brought down the price of PCs, telephone mainlines and cellular and Internet subscriptions. In such economies, it may be appropriate to undertake further research and micro-level analysis of consumer behaviour and choice preferences of citizens in relation to different telecommunication products beyond the confines of the variables employed in this study.

### The way forward

Internationally, a number of policies and strategies are available to support Africa's development towards realizing sustainable e-health usage. We have already alluded to the recent resolution of the World Health Assembly [4] and the health-for-all policy for the 21^st ^century [2] that underscore the potential role of ICT in health. The regional development and political forums such as the New Partnership for Africa's Development (NEPAD), sub-regional economic communities, regional development banks and the United Nations Economic Commission for Africa have elements in their policies and/or strategies encompassing ICT development. The Blair Commission for Africa advocates for massive investment in ICT and Internet connectivity [25] and there is a growing realization among bilateral and multilateral donor agencies of the need for supporting investments in ICT infrastructure and Internet connectivity in developing countries as an essential strategy for economic growth.

Therefore, the current policy environment for e-health growth internationally is very encouraging. With the passage of the World Health Assembly resolution [4], the health sector in the African Region can now lay claim to a legitimate basis for seeking internal and external resources to accelerate growth in this area.

**Figure 2:**
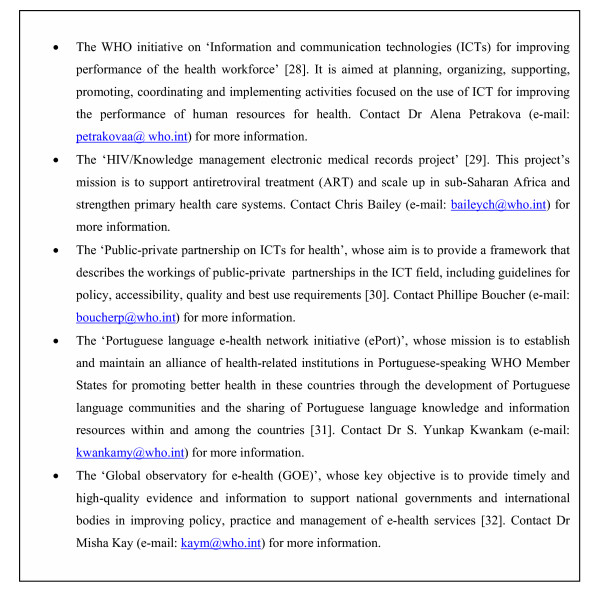
e-health-related initiatives/projects by WHO.

With regard to the creation of an enabling environment for equitable growth of e-health services as part of its stewardship role, each government in the Region should:

a. Develop a comprehensive policy and a legal and strategic framework to guide and nurture the growth of ICT, while at the same time protecting the welfare of its citizens. E-health should be deeply embedded within that framework, which should be developed in a participative manner using a multisectoral approach involving all stakeholders including development partners;

b. Invest in rural electrification in order to attract private investment in ICT in those areas;

c. Continue the pursuit of goals of universal access to adult literacy, primary and secondary education and primary health care;

d. Make the necessary investment in ICT infrastructure, including fixed phone lines installation, equipment (e.g. computers, servers, networks) and Internet connectivity in the entire health system (i.e. from the Ministry of Health (MoH) down to the level of community-based public health programmes), with special attention to rural areas;

e. Strengthen human capacities for judicious utilization of ICT at all levels of the national health system in pursuit of public health goals and objectives;

f. Embark on liberalization of the ICT industry to ensure competitive prices of ICT services, including e-health. However, unless there is intervention from the State and development partners, the inequities in access to computers and Internet networks (and hence to health-enhancing interventions) between the 'haves' and 'have-nots' are likely to widen the health care inequities and hence health inequalities in the Region;

g. Forge South-South and North-South partnerships to leverage public health information and expertise and e-based courses offered at various reputable institutions of higher learning;

h. Leverage the freely available health-related ICT resources such as:

• the '*biomedcentral.com*' that houses a large number of peer reviewed, online health-related journals [26];

• the Health InterNetwork Access to Research Initiative (HINARI) that provides free or very low-cost online access to 2800 major journals in biomedical and related social sciences to local, non-profit institutions in developing countries [27].

i. Tap into the e-health-related initiatives/projects that are at various stages of development by WHO contained in Figure 2.

## Conclusion

E-health offers unprecedented opportunities for improving equity in access to health-enhancing global public goods and health interventions. However, the African Region's transition to e-health faces a number of challenges: high adult illiteracy rates, low primary and secondary schools and tertiary institutions enrolment rates, dearth of ICT technical know-how, low per capita incomes, lack of ICT infrastructure and limited Internet connectivity. This calls for concerted domestic, complemented with external, investments in secular education, ICT equipment and infrastructure, e-health-related human resource capacities and Internet connectivity.

In order to improve access to health care, especially for the majority of Africans living in remote rural areas, there is urgent need to boost the availability and utilization of e-health services. Thus, universal access to e-health ought to be a vision for all countries in the African Region. Each country ought to develop a clear road map in a strategic e-health plan that will, over time, enable its citizens to realize that vision.

## Abbreviations

ICT – Information and communications technology

HINARI – Health InterNetwork Access to Research Initiative

ITU – International Telecommunications Union

NET – Internet connectivity

MDG – Millennium Development Declaration

NEPAD – New Partnership for Africa's Development

NHS – National Health Service

NPfIT – National Program for Information Technology

PCs – personal computers

UNDP – United Nations Development Programme

WHA – World Health Assembly

WHO – World Health Organization

## Competing interests

The author(s) declare that they have no competing interests.

## Authors' contributions

JMK, AS, DG, LHK and JS participated in the literature review, development of conceptual framework, data analysis and drafting of various sections of the manuscript. All authors read and approved the final manuscript.

## Pre-publication history

The pre-publication history for this paper can be accessed here:


